# Acute peritonitis caused by the acute pancreatitis of an ectopic pancreas in a jejunal duplication, in an adult with intestinal malrotation: a case report

**DOI:** 10.1186/s40792-023-01736-2

**Published:** 2023-08-28

**Authors:** Chihiro Yoshikawa, Kazuhiro Migita, Ichiro Yamato, Masato Ueno, Hisanori Kashizuka, Koichi Murakami, Hirofumi Ishikawa

**Affiliations:** Department of Surgery, Nara Prefecture Seiwa Medical Center, 1-14-16 Mimuro Sango-Cho Ikoma-Gun, Nara, 636-0802 Japan

**Keywords:** Ectopic pancreas, Intestinal duplication, Intestinal malrotation

## Abstract

**Background:**

Intestinal duplication and ectopic pancreas are two rare independent congenital anomalies. Few reports describe cases of patients with ectopic pancreas in an intestinal duplication causing acute peritonitis.

**Case presentation:**

A 31-year-old man was admitted to the hospital for epigastric pain. The patient was diagnosed with acute peritonitis caused by the acute pancreatitis of an ectopic pancreas in a jejunal duplication, with intestinal malrotation. The patient underwent the partial resection of the jejunum and Ladd’s procedure. The histopathological findings indicated ectopic pancreatitis in the jejunal duplication.

**Conclusions:**

We presented the case of acute peritonitis caused by the acute pancreatitis of an ectopic pancreas in a jejunal duplication in an adult with intestinal malrotation. Surgery is the primary treatment and is necessary for a definitive diagnosis.

## Background

Intestinal duplication is a rare congenital anomaly, which can be localized anywhere from the mouth to the anus [1]. Ectopic pancreatic tissue is commonly present in the gastrointestinal tract. Only 10 cases of an ectopic pancreas in an intestinal duplication have been reported. Herein, we reported the case of acute peritonitis caused by the acute pancreatitis of ectopic pancreas in a jejunal duplication in an adult with intestinal malrotation.

## Case presentation

**Fig. 1:**
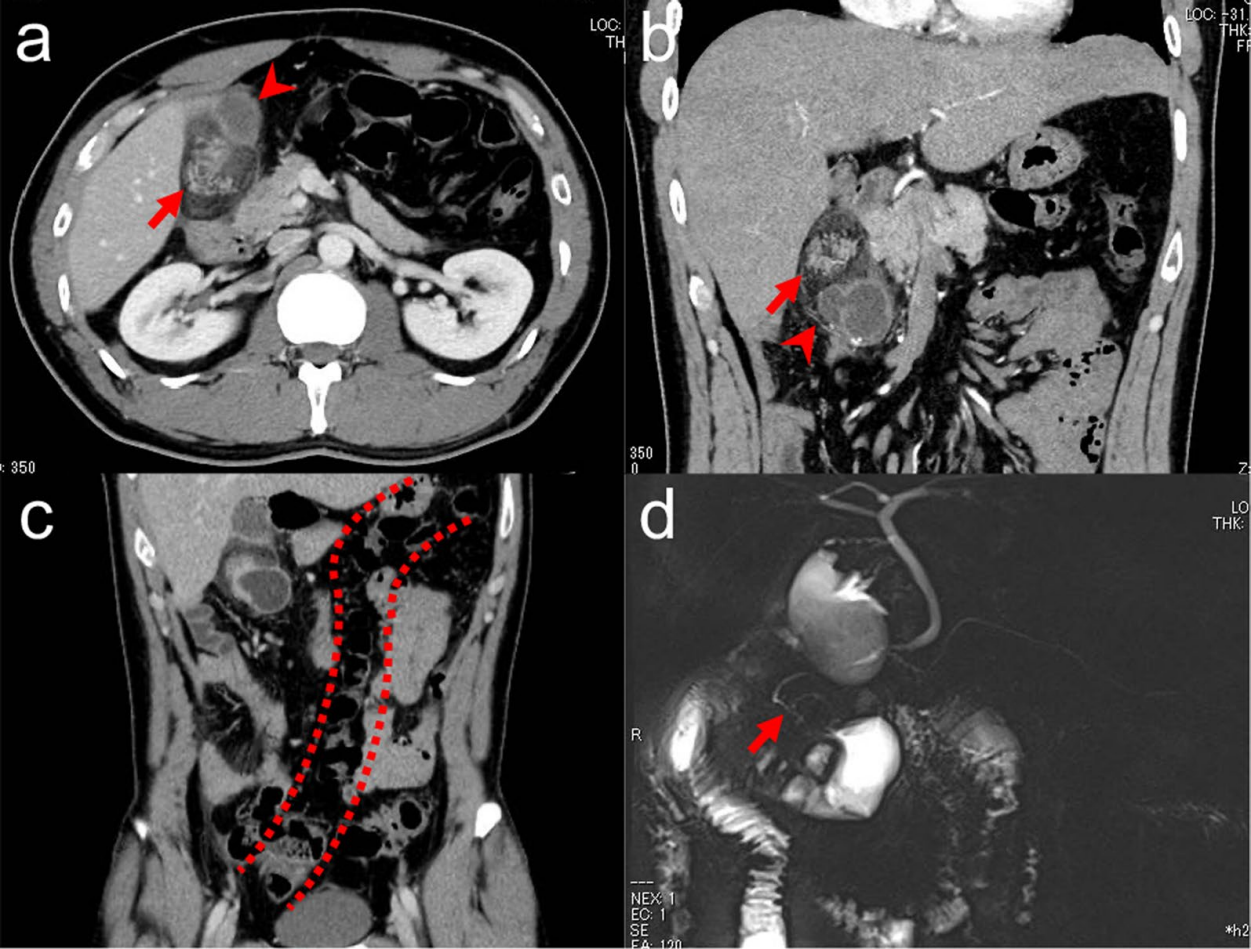
Abdominal contrast-enhanced computed tomography (CT) and magnetic resonance cholangiopancreatography (MRCP) on admission. **a** The axial CT image shows an oval 30 mm soft tissue mass with microlobulated margins (arrow) below the gallbladder (arrowhead). **b** The coronal CT shows an oval soft tissue mass with microlobulated margins, suggestive of an ectopic pancreas (arrow) and a cystic lesion with an enhanced thick wall that communicated with the jejunum (arrowhead). **c** The coronal CT image shows an ascending colon that runs up the midline. **d** MRCP shows an ectopic pancreatic duct (arrow) without communication to the normal pancreatic duct. The duct drained into the jejunal duplication

A 31-year-old man with no related medical history visited his general practitioner because of epigastric pain. Acute cholecystitis was suspected, and the patient was referred to the surgical department of our hospital. On admission, the vital signs were as follows: blood pressure, 124/70 mmHg; pulse, 70 beats/min; and temperature, 36.5 °C. The physical findings included right upper quadrant pain, negative Murphy sign, and no rebound pain. Blood tests revealed the following elevated inflammatory markers; white blood cell count, 9300/μL (3100–8400/μL); C-reactive protein level, 12.1 mg/dL (< 0.3 mg/dL); total bilirubin level, 2.2 mg/dL (0.4–1.5 mg/dL), and gamma glutaryl transferase level, 87 U/L (13–64 U/L). Contrast-enhanced abdominopelvic computed tomography (CT) revealed a 30-mm oval soft tissue mass with microlobulated margins below the gallbladder. The density of the mass was similar to the pancreas (Fig. 1a). In addition, CT revealed a cystic lesion adjacent to the mass with a thick enhanced wall, which communicated with the jejunum (Fig. 1b). These findings suggested an ectopic pancreas and a jejunal duplication. CT also showed that the ascending colon ran up the midline, suggesting that the patient had intestinal malrotation (Fig. 1c). Magnetic resonance cholangiopancreatography (MRCP) revealed an ectopic pancreatic duct without communication to the normal pancreatic duct draining into the jejunal duplication (Fig. 1d). Based on these findings, the patient was diagnosed with acute peritonitis due to the acute pancreatitis of ectopic pancreas in a jejunal duplication and intestinal malrotation. The acute pancreatitis was classified as mild according to the revised Atlanta classification of acute pancreatitis; the patient exhibited neither organ failure nor local complication [2].

**Fig. 2:**
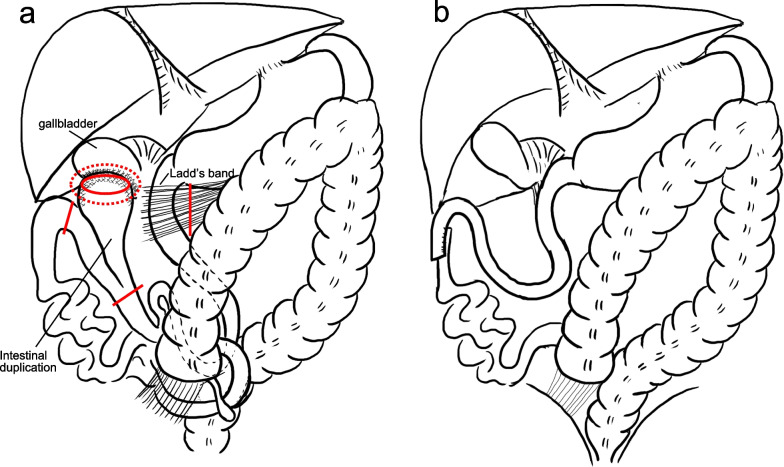
Scheme of intraoperative findings. **a** Preoperative image. Intra-abdominal abscess was observed between the tip of duplication and the gallbladder (the area that has been dotted circled). A 12-cm jejunal duplication was found on the opposite side of the mesentery, located 100 cm distal to the descending part of the duodenum. The tip of the duplication was swollen. Additionally, cord-like tissue was found between the duodenum and the ascending colon. The ligament of Treitz was absent, and the cecum and ascending colon were not fixed to the retroperitoneum. Partial resection of the jejunum including the jejunal duplication was performed. The Ladd’s procedure, including release of the Ladd’s band, repositioning of the duodenal flexure and appendectomy, was performed (red line). The ectopic pancreas is in the tip of the duplication (the area that has been circled). **b** Postoperative image

The patient was treated with antibiotics his symptoms and blood test parameters improved in 5 days. However, contrast-enhanced CT revealed indistinct margins and increased fat density around the ectopic pancreas and jejunal duplication, suggesting worsening peritonitis. Thus, the patient was scheduled for surgery. Although the surgery indicated as a laparoscopy, it was converted to open laparotomy because of inflammation and adhesions in the right upper quadrant of the abdominal cavity. An intra-abdominal abscess was observed between the tip of duplication and the gallbladder. Adhesions between the gallbladder and small intestine were dissected, and the abscess cavity was opened. A 12-cm jejunal duplication was observed on the opposite side of the mesentery, located 100 cm distal to the descending part of the duodenum. The tip of the duplication was swollen. Additionally, cord-like tissue was found between the duodenum and the ascending colon. The ligament of Treitz was absent, and the cecum and ascending colon were not fixed to the retroperitoneum (Fig. 2a). Partial resection of the jejunum, including the jejunal duplication, was performed. The Ladd’s procedure, including release of the Ladd’s band, repositioning of the duodenal flexure, and appendectomy, was performed (Fig. 2b). The operation time was 176 min and the intraoperative blood loss was 5 mL. The postoperative course was uneventful, and the patient was discharged on postoperative day 11.

**Fig. 3:**
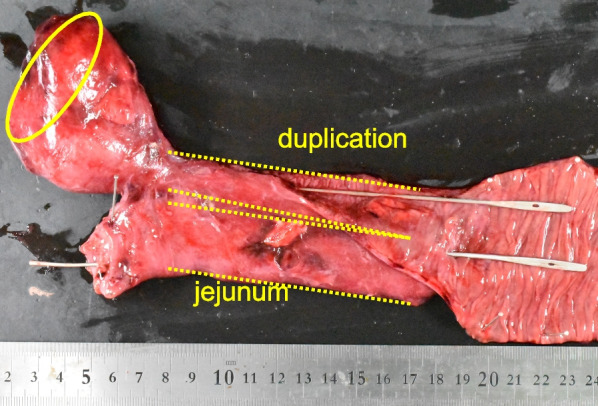
Macroscopic findings. The jejunal duplication bifurcates from the jejunum in a T-shaped manner, and communicates with the lumen of the normal jejunum. The tip of the duplication is swollen like a mass. The ectopic pancreas is in the tip of the duplication (the area that has been circled)

**Fig. 4:**
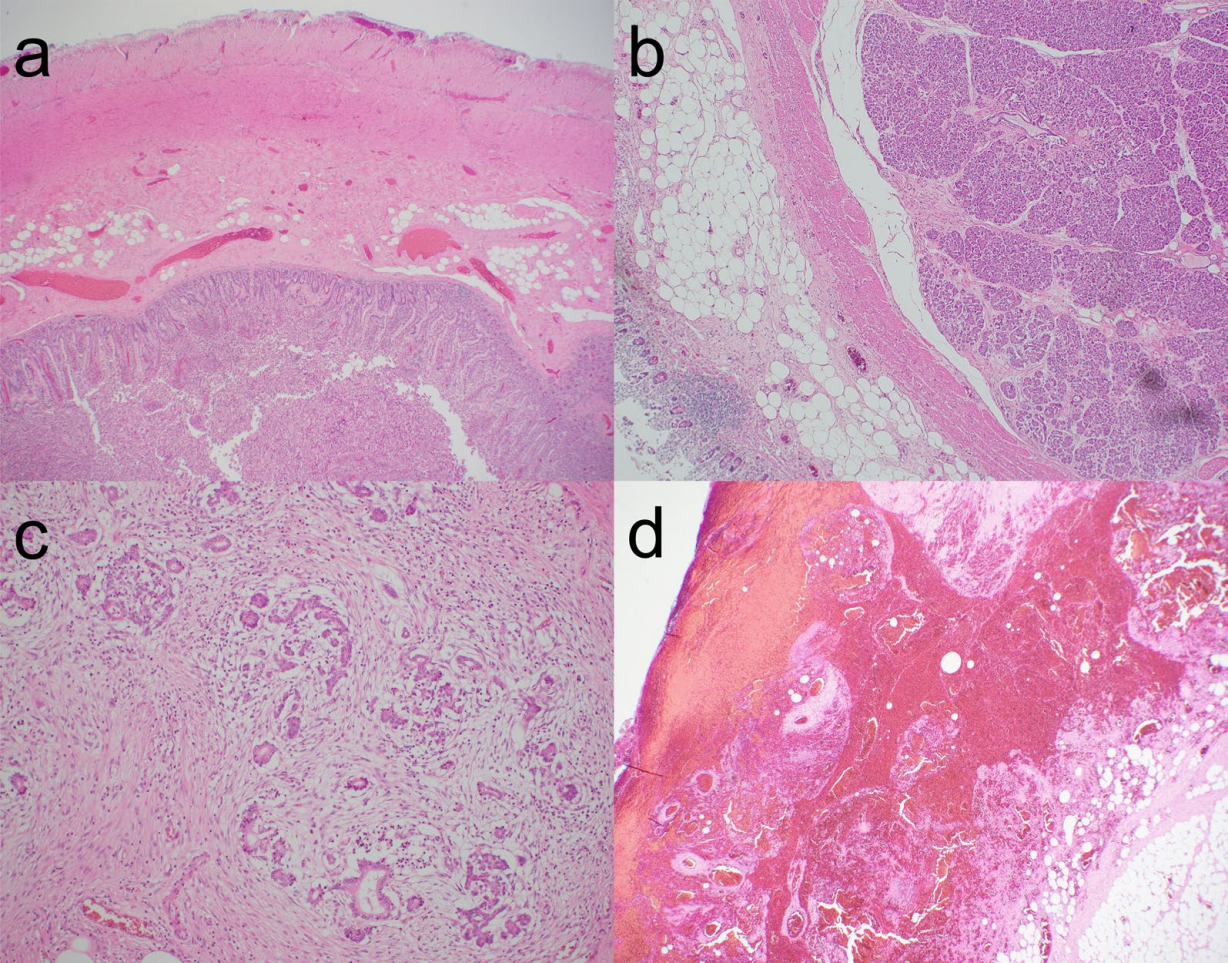
Microscopic findings. **a** Jejunal duplication (hematoxylin–eosin staining, ×40). The jejunal duplication comprises intestinal mucosa, submucosa, muscular, and serosal layers. **b** Ectopic pancreatic tissue (hematoxylin–eosin staining, ×40). Ectopic pancreatic tissue with acini and ducts is observed in the adipose tissue between the muscular and serosal layers at the tip of the duplication. **c** Chronic pancreatitis (hematoxylin–eosin staining, ×40). Part of the ectopic pancreatic tissue exhibits chronic pancreatitis with the proliferation of fibroblasts and lymphocytic infiltration. **d** Hemorrhagic necrosis (hematoxylin–eosin staining, ×100). Hemorrhagic necrosis is observed in the serosa at the tip of the duplication, and pancreatic tissue is not detected

The macroscopic findings of the resected specimen revealed jejunal duplication bifurcated from the jejunum in a T-shaped manner and communicating with the lumen of the normal jejunum (Fig. 3). Microscopically, intestinal mucosa, submucosa, and muscular and serosal layers were detected in the jejunal duplication (Fig. 4a). Ectopic pancreatic tissue with acini and ducts was observed in the adipose tissue between the muscular and serosal layers at the tip of the duplication (Figs. 3b, 4b). Part of the ectopic pancreatic tissue exhibited chronic pancreatitis with the proliferation of fibroblasts and lymphocytic infiltration (Fig. 4c). Hemorrhagic necrosis was observed in the serosa at the tip of the duplication, and pancreatic tissue was not identified, probably because of the strong inflammation (Fig. 3d).

## Discussion

We reported an extremely rare case of acute peritonitis caused by the acute pancreatitis of an ectopic pancreas in a jejunal duplication with intestinal malrotation. Intestinal duplication is a rare congenital anomaly that can affect any part of the gastrointestinal tract. The most frequent location of intestinal duplication is the ileum (31.5%), followed by the ileocecal valve (30.2%), duodenum (9.6%), stomach (8.2%), jejunum (8.2%), colon (6.8%), and rectum (5.5%) [3]. An ectopic pancreas, which is a relatively rare congenital condition, involves pancreatic tissue lacking anatomical or vascular continuity with the normal pancreas. Ectopic pancreas can occur in the gastrointestinal tract, biliary system, liver, spleen, lung, mediastinum, and brain; the most common location is the stomach followed by the duodenum (25%–35%) and jejunum (16%) [4–7]. Most cases of ectopic pancreas are identified incidentally during surgery or upon autopsy. Autopsy reports indicate that the prevalence of ectopic pancreas range from 0.6% to 13.7% [8].

Table 1 summarizes 11 cases of ectopic pancreas in intestinal duplication, including the present case [9–18]. The median age of the eight male patients and three female patients was 35 (15–69) years. The intestinal duplications were located in the jejunum in five cases, the stomach in three cases, the ileum in two cases, and other in one case. Patients with intestinal duplications exhibit various symptoms, including abdominal distension, abdominal pain, hematemesis, and an abdominal painless mass. The symptoms are related to the location, size, and shape of the intestinal duplication. The acute complications of intestinal duplications, such as bleeding, perforation, intestinal obstruction, and intussusception, are rare [19]. An ectopic pancreas is usually asymptomatic but can exhibit complications similar to normal pancreatic tissue, such as pancreatitis, abscess and pseudocyst formation, and malignant transformation [7, 8, 20, 21]. The most common symptoms in patients with ectopic pancreas include epigastric pain, abdominal fullness, and hematochezia [22]. Table 1 shows that 10 (90.9%) patients exhibited symptoms and jejunal duplication with ectopic pancreas was incidentally detected intraoperatively in 1 patient. Three patients (Patients 1, 2, and 8) exhibited symptoms related to the inflammation of the intestinal duplication, and three patients (Patients 4, 5 and 11) exhibited symptoms related to the inflammation of the ectopic pancreas. The inflammation of the intestinal duplication may be due to the stagnation in the blind pouch variety of duplication. However, the causes of inflammation in the ectopic pancreas are unknown. In our patient, the chief complaint was epigastric pain. The pathological findings revealed the highest inflammation in the serosal and the normal mucosal surfaces of the intestinal duplication. Hemorrhagic necrosis was observed in the serosa at the tip of the duplication. The pancreatic tissue could not be identified in the area where hemorrhagic necrosis was observed, possibly because the inflammation was sufficiently strong to cause the disappearance of the pancreatic tissue. The pathological findings suggested that this symptom was caused by the inflammation of the ectopic pancreas, although the patient had no episodes causing pancreatitis.

**Table 1:** Previous reports of ectopic pancreas in intestinal duplication in adults

No.	Author	Year	Sex	Age (years)	Location	Symptom	Condition	Preoperative diagnosis	Treatment
1	Mizumoto [9]	2009	Male	69	Jejunum	Abdominal pain	Inflammation	Fish bone perforation	Small bowel resection
2	Asano [10]	2009	Male	35	Ileum	Abdominal pain	Inflammation	Acute appendicitis	Small bowel resection
3	Shinha A [11]	2010	Female	30	Stomach	Abdominal pain	–	Duplication cyst and ectopic pancreas	Partial gastrectomy
4	Emoto [12]	2011	Male	20	Retroperitoneum	Abdominal pain Vomiting	Inflammation	Lymphangioma, mature teratoma or pancreatic pseudocyst	Cystectomy
5	Seita [13]	2014	Male	35	Jejunum	Abdominal pain	Inflammation	GIST	Small bowel resection
6	Gruzu S [14]	2016	Male	51	Jejunum	No symptoms	No symptoms	None (incidentally detected)	Small bowel resection
7	Passos [15]	2017	Male	38	Stomach	Abdominal pain Vomiting	Obstruction	Gastric duplication	Partial gastrectomy
8	Ito [16]	2020	Female	59	Jejunum	Abdominal pain	Inflammation	GIST	Small bowel resection
9	Wang TL [17]	2022	Male	29	Ileum	Abdominal pain Bloating	Obstruction	Intestinal obstruction and intussusception	Small bowel resection
10	Ye X [18]	2022	Female	15	Stomach	Vomiting	Obstruction	Gastric duplication and ectopic pancreas	ESD
11	Our case	2023	Male	31	Jejunum	Abdominal pain	Inflammation	Jejunal duplication and ectopic pancreas	Small bowel resection

GIST gastrointestinal stromal tumor; ESD endoscopic submucosal dissection

The preoperative diagnosis of intestinal duplication and ectopic pancreas may be difficult owing the rarity and absence of specific findings. CT may provide some informative findings, and endoscopic ultrasonography (EUS) may be useful for the intestinal segments that can be reached by an endoscope. CT typically shows a spherical duplication cyst as a thick-walled cystic lesion with enhancement of the inner lining and tubular duplication as an intestinal structure with a blind end [15]. The ectopic pancreas appears as a round or oval intramural soft tissue mass with smooth or microlobulated margins on CT [23]. On MRI, the ectopic pancreas generally appears isointense to the orthotopic pancreas in all the sequences, and MRCP may show a duct-like structure inside the lesion. MRI may help distinguish the ectopic pancreas from other submucosal lesions to help diagnose ectopic pancreas [24, 25]. Ectopic pancreas in intestinal duplications were diagnosed preoperatively in only three cases (27.3%), including the present case (Table 1); ectopic pancreas was diagnosed via CT in two patients and via EUS in one patient. In our patient, ectopic pancreas in intestinal duplications was diagnosed preoperatively based on the typical CT and MRI findings. The CT performed immediately after the appearance of symptoms may facilitate accurate diagnosis because the pancreatic structure in the ectopic pancreas can be preserved.

In the reported cases, the ectopic pancreas in the intestinal duplication was resected using endoscopic submucosal dissection (ESD), apart from l the duplication muscle layer in one case. ESD is a treatment option if the lesion is amenable to endoscopic resection and is adequately deep to be safely removed. In other cases, including the present case, small bowel resection or partial gastrectomy, including the intestinal duplication and the cyst, were performed. In general, complete resection is the treatment of choice for symptomatic, possibly malignant, and incidental cases of ectopic pancreas to prevent future complications.

Our patient also had intestinal malrotation. The intestinal malrotation was most likely not associated with the peritonitis in this case. The coexistence of intestinal malrotation with ectopic pancreas and intestinal duplication is extremely rare, and this was the first case to be reported to the best of our knowledge.

## Conclusions

We present a case of acute peritonitis caused by the acute pancreatitis of an ectopic pancreas in a jejunal duplication in an adult with intestinal malrotation. CT and MRI may be useful for the preoperative diagnosis of ectopic pancreas. Surgery is the primary treatment and is necessary for a definitive diagnosis. This report aimed to raise awareness and provide information regarding this rare case to help clinical management.

## Data Availability

Not applicable.

## Abbreviations

CT: Computed tomography
ESD: Endoscopic submucosal dissection
EUS: Endoscopic ultrasonography
MRCP: Magnetic resonance cholangiopancreatography
